# Commentary on: Peptidylglycine α-amidating Monooxygenase is Required for Atrial Secretory Granule Formation

**DOI:** 10.33696/cardiology.2.022

**Published:** 2021

**Authors:** Emil Daniel Bartels, Jens Peter Gøtze, Richard E. Mains, Betty A. Eipper

**Affiliations:** 1 Department of Clinical Biochemistry, Rigshospitalet, University of Copenhagen, Copenhagen, Denmark; 2 Department of Clinical Medicine, University of Copenhagen, Copenhagen, Denmark; 3 Department of Biomedical Sciences, University of Copenhagen, Copenhagen, Denmark; 4 Department of Neuroscience, University of Connecticut Health Center, Farmington CT USA; 5 Department of Molecular Biology & Biochemistry, University of Connecticut Health Center, Farmington CT USA

## Abstract

The electron-dense spherical granules found in the perinuclear region of atrial myocytes store and release both proatrial and probrain natriuretic peptides (proANP and proBNP, respectively). Mature ANP and BNP produce vasodilation and natriuresis and inhibit the renin-angiotensin and sympathetic nervous systems. Although neither ANP nor BNP is a-amidated, Peptidylglycine a-Amidating Monooxygenase (PAM), an integral membrane enzyme known to catalyze the a-amidation of peptidylglycine precursors, is the major atrial granule membrane protein. Selective deletion of PAM from cardiomyocytes impairs their ability to store proANP, resulting in an increase in proANP secretion. Exogenous expression of active or inactive PAM protein restores the ability of atrial myocytes to store proANP, leading to the suggestion that PAM functions as a cargo receptor for newly synthesized proANP.

## The Basic Science

**Figure 1:**
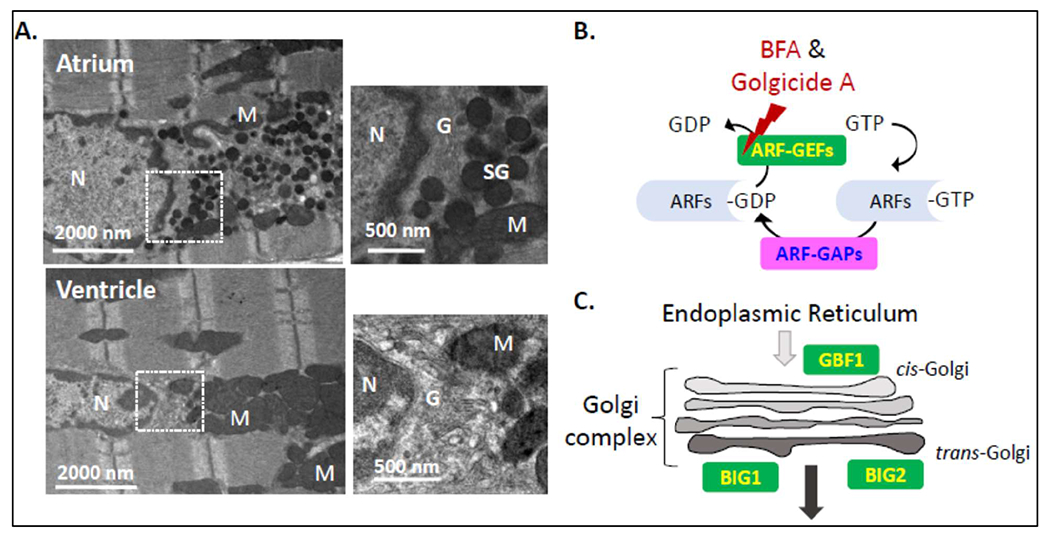
**A.** Transmission electron microscopic images highlight the secretory granules (SG) that accumulate near the Golgi (G) complex in atrial, but not in ventricular, myocytes. In both atrial and ventricular myocytes, the Golgi complex abuts the poles of the nucleus (N) and mitochondria (M) are prevalent. **B.** ARF proteins are active when GTP is bound and inactive when GDP is bound; ARF GDP/GTP Exchange Proteins (GEFs) and ARF-GTPase Activating Proteins (GAPS) control their activation/inactivation. Brefeldin A (BFA) and Golgicide A (GCA) inhibit selected Golgi-localized ARF-GEFs. **C.** GBF1, the only ARF-GEF localized to the *cis*-Golgi is inhibited by both BFA and GCA; BIG1 and BIG2, ARF-GEFs localized to the *trans*-Golgi and endocytic pathway are inhibited by BFA, but not by GCA.

The electron-dense spherical granules found in atrial myocytes are one of the most obvious features distinguishing these cells from ventricular myocytes (Figure 1A). In mouse atrial myocytes, granules containing atrial natriuretic peptide (ANP) and brain natriuretic peptide (BNP) accumulate at one pole of the nucleus, adjacent to a voluminous Golgi complex (Figure 1A) [1,2]. Atrial granules, with their electron dense core and surrounding membrane, look much like the granules that store insulin in islet β-cells and vasopressin in magnocellular hypothalamic neurons. N-terminal signal sequences guide newly synthesized proANP, proinsulin and provasopressin into the lumen of the endoplasmic reticulum (ER), where essential disulfide bonds are formed.

Instead of accumulating near the plasma membrane, ready for release in response to appropriate stimuli, atrial granules remain closely associated with the Golgi complex [2]. The precursors to insulin and vasopressin must undergo a series of prohormone convertase mediated cleavages as they traverse the Golgi complex, exit the *trans*-Golgi and enter immature secretory granules. Prohormone convertases are not expressed in cardiomyocytes and proANP is stored intact, undergoing Corin-mediated cleavage at the time of secretion [3,4]. Stretch is the major stimulant of ANP release, but endothelin and catecholamines, acting through their G protein coupled receptors, also stimulate ANP release. Calcium entry plays a major stimulatory role in the release of insulin and vasopressin, but has a mild inhibitory effect on ANP secretion [5]. Brefeldin A, a fungal metabolite that disrupts vesicular trafficking by inhibiting Golgi-localized GDP/GTP exchange factors (GEFs) for several ADP ribosylation (ARF) proteins (Figures 1B and 1C), generally inhibits basal secretion. However, pioneering studies on the secretion of proANP by cannulated rat atria and primary cultures of atrial myocytes identified Brefeldin A as a stimulator of basal proANP secretion [6].

**Figure 2:**
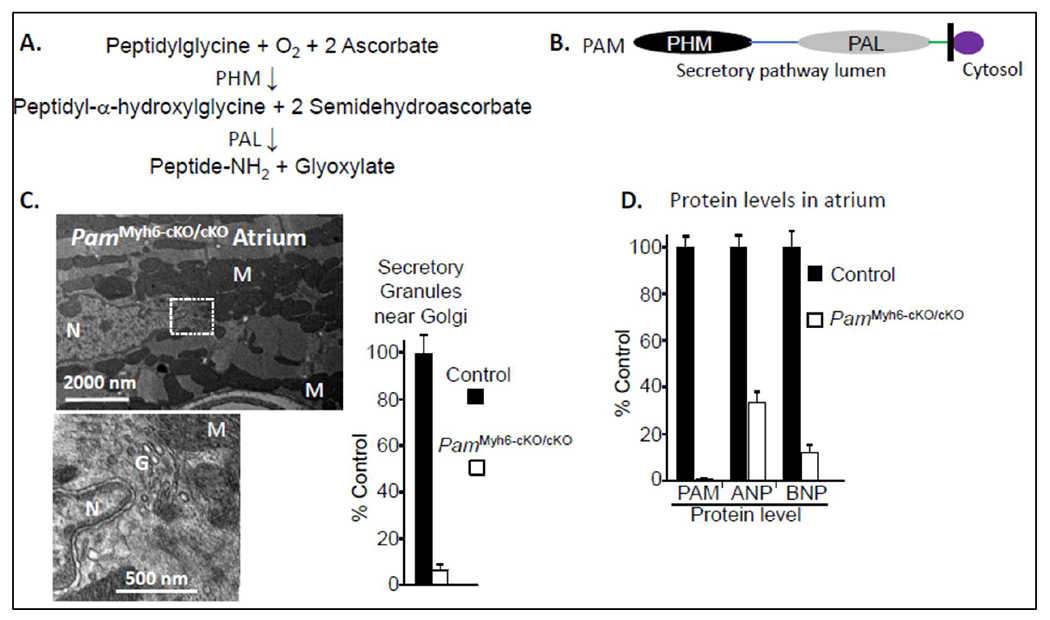
PAM knockout mice. The reaction catalyzed by PAM is outlined (**A**), along with the major form of PAM in humans (**B**) and its two enzymatic domains, PHM and PAL (peptidylglycine a-hydroxylating monooxygenase and peptidyl-a-hydroxyglycine a-amidating lyase; E.C. 1.4.17.3 and 4.3.2.5). **C.** Transmission electron micrographs of the *Pam*^Myh6-cKO/cKO^ adult atrium; secretory granules are rarely seen near the peri-nuclear Golgi or elsewhere. Quantitative data comparing the number of Golgi-localized secretory granules observed in control vs. *Pam*^Myh6-cKO/cKO^ atria revealed a nearly 20-fold decrease (Figure 1A). **D.** Immunoblot analyses of adult atria established near-total ablation of PAM and major loss of proANP and proBNP in *Pam*^Myh6-cKO/cKO^ atrium. Data replotted from [13].

Atrial granules contain high levels of proANP and proBNP [1,7]. Surprisingly, the major protein in their membranes is Peptidylglycine a-Amidating Monooxygenase (PAM) [8], a Type 1 integral membrane enzyme that catalyzes the two-step conversion of peptidylglycine substrates into amidated product peptides plus glyoxylate (Figures 2A, and 2B). The copper and ascorbate-dependent monooxygenase domain of PAM (PHM) converts peptidylglycine substrates into peptidyl-a-hydroxyglycine-containing intermediates. The zinc-dependent lyase domain (PAL) converts the penultimate residue of the peptide into an amidated product, releasing glyoxylate (reviewed in [9,10]) (Figures 2A). While many peptides (e.g. vasopressin, gastrin, neuropeptide Y, thyrotropin releasing hormone) are inactive until amidated by PAM, neither proANP, ANP, proBNP, nor BNP is amidated. Early studies revealed high levels of PAM in the atrium, with transient expression of PAM in the ventricles during development [11], but its function in cardiac tissue has remained a mystery for 40 years.

Genetic manipulations that eliminate PAM expression in mice, flies, and zebrafish are not compatible with life [10]. To explore the role of PAM in the heart, mice with a floxed allele of PAM (*Pam*^cKO/cKO^) were mated to mice in which the expression of Cre-recombinase was driven by the cardiomyocyte-specific myosin heavy chain 6 (Myh6) promoter (*Pam*^Myh6/+;cKO/cKO^). Mice unable to express PAM in their cardiomyocytes (*Pam*^Myh6-cKO/cKO^) are viable [12]. Electron microscopy revealed the almost total absence of secretory granules in the atria of *Pam*^Myh6-cKO/cKO^ mice (Figure 2C), with few other changes in their ultrastructure [13]. Atrial levels of proANP and proBNP protein declined in the absence of PAM (Figure 2D).

**Figure 3.**
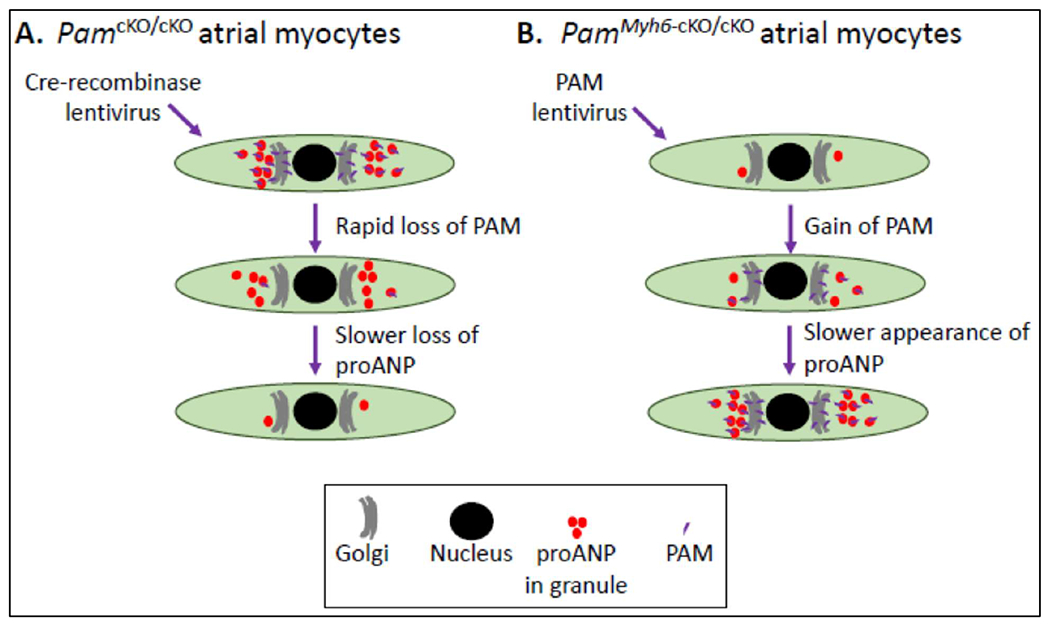
Necessary and sufficient. Experimental strategies used to demonstrate the essential role played by the PAM protein in proANP storage (**A**) and rescue of proANP storage in atrial myocytes lacking PAM (*Pam*^Myh6-cKO/cKO^) in response to expression of exogenous PAM; surprisingly, even PAM in which the mutation of a single active site residue eliminated its monooxygenase activity (PAM/Met314Ile) was still capable of restoring proANP storage. (**B**). Data reported in [13].

Using primary cultures of atrial myocytes from *Pam*^cKO/cKO^ mice and lentiviral delivery of Cre-recombinase, we established that loss of PAM caused a reduction in proANP content (Figure 3A) [13]. Using primary cultures of atrial myocytes from *Pam*^Myh6-cKO/cKO^ mice and lentiviral delivery of PAM, we demonstrated that expression of PAM in cells which had not previously expressed PAM was sufficient to rescue proANP content to near normal (Figure 3B). PAM lacking one of its essential copper binding sites is enzymatically inactive; strikingly, expression of inactive PAM also restored the proANP content of *Pam*^Myh6-cKO/cKO^ atrial myocytes to near normal (Figure 3B) [13].

**Figure 4:**
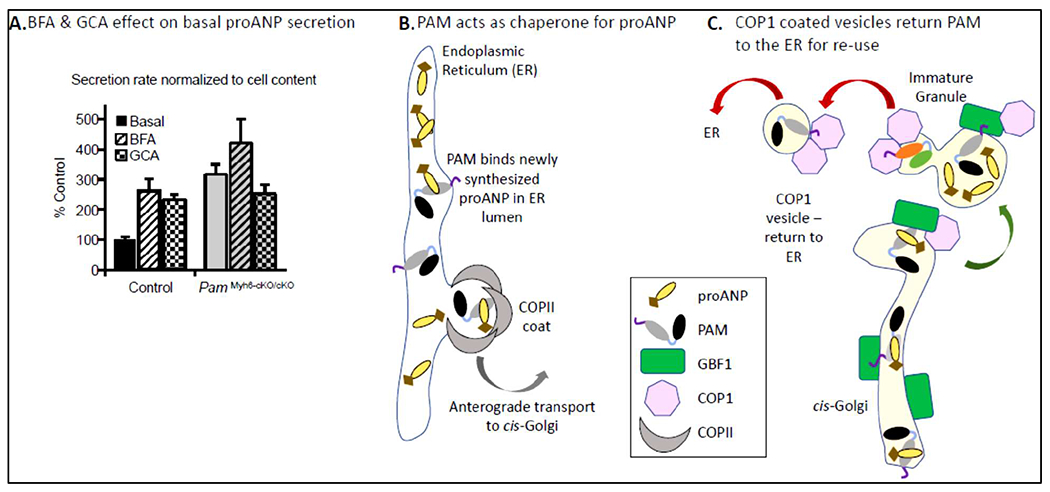
Proposed mechanism. **A.** The effects of BFA and GCA on the basal secretion of proANP by primary cultures of control and *Pam*^Myh6-cKO/cKO^ atrial myocytes was assessed. **B.** In our working model, the luminal domains of PAM bind newly synthesized proANP, facilitating its delivery to the *cis*-Golgi; PAM/Met314Ile can perform this same function. **C.** The delivery of proANP to nascent granules is facilitated by PAM; the return of PAM to the ER for re-use requires GBF1. In the absence of PAM, the basal secretion of proANP increases three-fold. Data from [13] depicted in diagrammatic form.

Neither proANP mRNA levels nor proANP biosynthesis differed in wildtype and *Pam*^Myh6-cKO/cKO^ atrial myocytes. However, a 3-fold increase in the basal secretion of proANP was observed in *Pam*^Myh6-cKO/cKO^ atrial myocytes, identifying increased secretion as a major cause of the observed decrease in proANP storage (Figure 4A). Consistent with earlier studies, BFA produced a 3-fold increase in the basal secretion of proANP by control atrial myocytes (i.e. a failure of storage); in contrast, BFA had no significant effect on proANP secretion by atrial myocytes lacking PAM. Golgicide A, which inhibits the GDP/GTP exchange activity of the only *cis*-Golgi localized ARF-GEF (GBF1) without affecting the *trans*-Golgi localized ARF-GEFs (Figure 1C), mimicked the effects of BFA (Figure 4A). GBF1 plays an essential role in the formation and trafficking of the COPI-coated vesicles that return cargo from the *cis*-Golgi to the ER. Taken together, our data support a model in which the PAM protein interacts directly with newly synthesized proANP (Figure 4B) [14], facilitating its transfer from the ER to the *cis*-Golgi and nascent granules (Figure 4C). With an ~30-fold molar excess of proANP over PAM, GBF1-dependent recycling of PAM plays an essential role in sustaining the efficient delivery of proANP to nascent granules [8]. The activity of its monooxygenase domain is not essential for PAM to play this role. As for several other secretory pathway cargo proteins, the successful storage of large amounts of proANP in granules requires the recycling of its chaperone [15]. A detailed discussion of PAM trafficking in the secretory and endocytic pathways and a comparison of atrial granules to lysosome-related organelles can be found in the review by Back et al. [9].

## The Clinical Implications

The lack of atrial secretory granules and reduced serum levels of ANP seen in *Pam*^Myh6-cKO/cKO^ mice raise several questions about the consequences of PAM deficiency for the paracrine and endocrine roles of ANP. First and foremost, complete lack of ANP or its principal receptor (NPR1, also designated NPR-A) in mice leads to salt-dependent hypertension and hypertrophy with concomitant fibrosis in the heart [16,17]. The apparent lack of a similar cardiovascular phenotype in the *Pam*^Myh6-cKO/cKO^ [12] suggests that the increased basal secretion of proANP, which is thought to occur via a Golgi bypass pathway in isolated *Pam*^Myh6-cKO/cKO^ atrial cardiac myocytes, is sufficient to counteract these changes. Neither plasma levels of BNP nor secretion of BNP by *Pam*^Myh6-cKO/cKO^ atrial myocytes in culture has been assessed, but atrial proBNP was even more depleted than proANP [12,13]. BNP is expressed in both atrial and ventricular myocytes in mice (in a ratio of approx. 1:1) [18]; secretory granules are rarely seen in adult ventricular myocytes, consistent with the fact that ventricular secretion of BNP is constitutive (not regulated). The consequences of the absence of atrial granules in *Pam*^Myh6-cKO/cKO^ mice may first manifest itself during stress-induced, pulsatile release of natriuretic peptides in response to disease, e.g. increased atrial stretch or ischemia, as previously described [19,20].

In animal models of ischemia-reperfusion injury, infusion of ANP or BNP has been shown to reduce the area at risk and infarct size [21] and both ANP and BNP are used clinically to treat cardiac disease [22,23]. Natriuretic peptides have been pursued as potential therapeutics for acute heart failure, although with mixed results due to side effects [24,25]. The clinical use of peptides as drugs may work best without the severe hemodynamic lability seen in heart failure. For example, resistant/essential hypertension may be an ideal diagnosis to begin a trial of natriuretic peptide treatment [26,27]; the cardio-specific PAM knockout models could be an ideal animal model to evaluate the efficacy of peptide treatment, as the endogenous hormonal axis is not capable of functioning normally. Notably, it is well known that mutations in Corin, the key processing enzyme that generates bioactive ANP and BNP from their precursors, are strongly associated with hypertension [28,29]. Whether the absence of PAM affects the ability of Corin to cleave pronatriuretic peptides released from the classical or Golgi bypass pathways has not yet been determined. Finally, whether the lack of pronatriuretic-peptide-containing granules will increase infarct size seems worthy of further mechanistic studies.

The production and release of natriuretic peptides is markedly upregulated during hormonal, hemodynamic, or oxygen-related cardiac stress [22]. However, it is not yet known how PAM deficiency affects the compensatory upregulation seen in hypertension, heart failure, and atrial fibrillation. PAM deficiency has been implicated in a number of human ailments, notably type II diabetes and hypertension [30–33]. Additional important aspects to address include the effects of lack of PAM on Corin trafficking and on BNP processing and secretion. Physiological and mechanistic studies in the cardio-specific PAM knock-out mouse model should prove to be of great value for better understanding the interplay between ANP and BNP in disease and elucidating how changes in the pulsatile release of natriuretic peptides affects the heart and circulation under stress.
